# Panoramic quality assessment tool for investigator initiated trials

**DOI:** 10.3389/fpubh.2022.988574

**Published:** 2022-09-13

**Authors:** Wenwen Lv, Tingting Hu, Jiayuan Jiang, Tiantian Qu, Enlu Shen, Jiacheng Duan, Xin Miao, Weituo Zhang, Biyun Qian

**Affiliations:** ^1^Hongqiao International Institute of Medicine, Shanghai Tongren Hospital and School of Public Health, Shanghai Jiao Tong University School of Medicine, Shanghai, China; ^2^Department of Cardiothoracic Surgery, Xinhua Hospital, School of Medicine, Shanghai Jiao Tong University, Shanghai, China; ^3^Department of Orthopaedic Surgery, Shanghai Jiaotong University Affiliated Sixth People's Hospital, Shanghai, China; ^4^Shanghai Clinical Research Promotion and Development Center, Shanghai Shenkang Hospital Development Center, Shanghai, China

**Keywords:** investigator initiated trials, quality assessment, tool, quality attributes, structural model

## Abstract

**Objectives:**

Quality can be a challenge for Investigator initiated trials (IITs) since these trials are scarcely overseen by a sponsor or monitoring team. Therefore, quality assessment for departments managing clinical research grants program is important and urgently needed. Our study aims at developing a handy quality assessment tool for IITs that can be applied by both departments and project teams.

**Methods:**

The framework of the quality assessment tool was developed based on the literature studies, accepted guidelines and the Delphi method. A total of 272 ongoing IITs funded by Shanghai non-profit organizations in 2015 and 2016 were used to extract quality indexes. Confirmatory factor analysis (CFA) was used to further evaluate the validity and feasibility of the conceptual quality assessment tool.

**Results:**

The tool consisted of 4 critical quality attributes, including progress, quality, regulation, scientificity, and 13 observed quality indexes. A total of 257 IITs were included in the validity and feasibility assessment. The majority (60.29%) were Randomized Controlled Trial (RCT), and 41.18% were multi-center studies. In order to test the validity and feasibility of IITs quality assessment tool, CFA showed that the model fit the data adequately. (CMIN/DF = 1.868, GFI = 0.916; CFI = 0.936; TLI = 0.919; RMSEA = 0.063; SRMR = 0.076). Different types of clinical studies fit well in the tool. However, RCT scored lower than prospective cohort and retrospective study in enrollment progress (7.02 vs. 7.43, 9.63, respectively).

**Conclusion:**

This study established a panoramic quality assessment tool based on the Delphi method and CFA, and the feasibility and effectiveness of the tool were verified through clinical research examples. The use of this tool can help project management departments effectively and dynamically manage research projects, rationally allocate resources, and ensure the quality of IITs.

## Introduction

Investigator initiated trials (IITs) complement the industry-sponsored trials (ISTs), optimize existing therapies or treatment approaches and attempt to answer clinical problems (1, 2). In addition, IITs do not only facilitate a better understanding of disease domain and drug effect, but help translate academic research into clinical practice. Recent literature also reported that IITs have changed the practice of medicine (3).

Industry-sponsored trials have rigorous monitoring and auditing to ensure the authenticity and reliability of data, while IITs often lack resources and may not have similar quality checks (4, 5). IITs are equally important to ISTs as they explore the use of marketed drugs for new indications and clinical diagnosis or treatment effect comparison (4). Thus the quality of IITs should be taken seriously (6). For hospitals, an alternative quality management system could be adopted to alleviate regulatory pressures (7), and a comprehensive and feasible quality assessment tool is also urgently required as IITs faced challenges both in design and conduct.

Over the past decade, more and more studies emphasized on the importance of methodology and the quality of research report, both in IITs and ISTs (8, 9). Several publications highlighted that risk-adapted monitoring was important for quality control and sufficient to identify critical questions in the conduct of clinical trials (10, 11). Quality assessment tools for research design or reporting were abundant, while few for operation and funding decision (12). The Risk-Based Monitoring Toolbox of European Clinical Research Infrastructure Network provided information on tools available for risk assessment, monitoring and study conduct. The toolbox was mainly created following literature review or surveys, and was a collection of risk-based tools and strategies (13). Take RACT and ADAMON as examples. In 2013, TransCelerate BioPharma, an independent non-profit organization, developed a Risk Assessment and Categorization Tool (RACT), which many biopharmaceutical companies have used to assess the risk level of clinical trials before the start of the trial as well as regular checkups. Despite that the RACT offered a very useful methodology for risk assessments of study level, it had some weaknesses when used to evaluate IITs. The RACT was prone to subjectivity and lacked important categories (14). Adapters Monitoring (ADAMON)^1^ risk scale can be used for IITs, but it's just a tool for assessing the required amount of on-site monitoring, not whole quality. Furthermore, Patwardhan et al. (15) drew up a checklist consisting of various criteria that were essential in IITs documentation, while not suitable to assess large-scale IITs. Systematic study about quality assessment tools designed for large-scale IITs was rare. The main goal of this study is to develop an operational quality assessment tool for IITs to enhance the quality of clinical research, ensure the safety of subjects and the authenticity and reliability of data, and avoid waste of resources.

## Materials and methods

### Index system construction

The quality indicators were extracted based on the following three principles. The indicators can be (1) used to evaluate the quality of clinical research (2) relatively simple and easy to understand, and (3) easily operated to ensure smooth progress of quality assessment. The preliminary quality indicators and the basic framework of IITs were firstly developed through indices development committee discussion, focus group interview, reviewing literature studies and accepted guidelines, including International Council on Harmonization E6 Guidance Revision 2 [ICH E6(R2)] (16), United States Food and Drug Administration (US FDA) (17), European Medicines Agency (EMA) (18), SPIRIT 2013 Statement (19), and CONSORT 2010 statement (20). The indices development committee that developed the IITs indices included various roles in clinical researches: those were a clinical expert, two statisticians, a project manager (PM), a data manager (DM), two clinical research associates (CRA), a senior research manager and a financial expert, all staffs have received GCP training.

**Figure 1:**
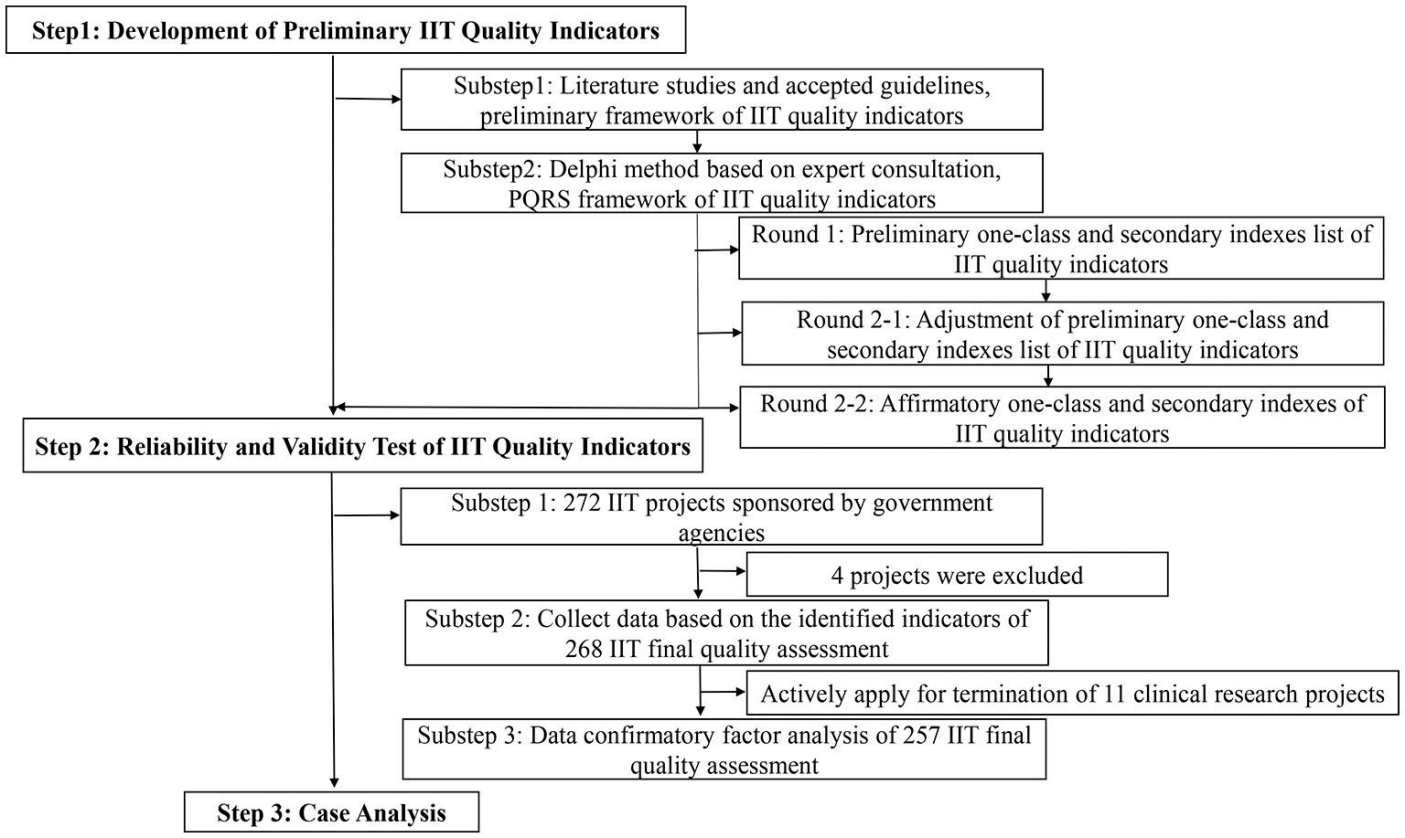
Flowchart of development and validation of quality assessment tool for IITs.

Investigator initiated trials should follow GCP principles to ensure protection of the trial subjects and assures quality and credibility of the data obtained. We used focus group interviews to collect and build critical attributes of IITs that the funding agencies like NIH, clinical research organizations and hospitals is most concerned about. Finally, four critical attributes of IITs were obtained, namely: progress, quality, regulation, and scientificity. We further expanded indices according to four critical attributes of IIT by referring to literature and accepted guidelines (Table 1). The importance and feasibility of the indicators were further evaluated through two rounds of the Delphi method. We consulted more than 20 experts with senior titles in each round, including clinical research methodologists, research managers, and clinicians engaged in clinical research. Furthermore, this research used confirmatory factor analysis (CFA) and structural equation modeling as tools to evaluate the structural validity of the index system (Figure 1).

**Table 1:** Framework of quality properties and indexes of quality assessment tool for IITs.

**Quality properties**	**Quality indexes**	**Description**	**Measurement**
Progress	Overall progress	The research is in different recruitment stages: not enrolled, enrolled subjects, enrolled completed, follow-up completed.	Quantitative data
	Enrollment progress	Enrolled subjects/Planned enrolled subjects	Quantitative data
	Budget implementation rate	Budget implementation/Planned budget	Quantitative data
Quality	Study protocol compliance	The actual implementation of the inclusion/exclusion criteria, grouping/interventions, randomization (if have), blinding (if have), primary and secondary endpoints shall be in accordance with the study protocol	□ Yes □ No □ Can't answer □ Not applicable
	Data management	Electronic data management system, critical data traceability, integrity and accuracy.	□Yes □ No □ Can't answer □ Not applicable
	Subject management	Subjects were followed up as planned, dropout rate is <20%/ lower than the dropout rate specified in the study protocol.	□ Yes □ No □ Can't answer □ Not applicable
	Quality control	Standards and SOPs of drug, device and sample management, quality control plan, quality assurance plan, independent data monitoring committee (if have), independent endpoint review committee (if have)	□ Yes □ No □ Can't answer □ Not applicable
Regulation	Ethical approval	Ethical approval documents, protocol amendments must be submitted to the IRB and must be approved by the IRB before they can be implemented, submit progress reports or a final report to the IRB.	□ Yes □ No □ Can't answer □ Not applicable
	Subject safety	Standard AE/SAE recording, SAE reporting and emergency rescue procedure.	□Yes □ No □ Can't answer □ Not applicable
	Informed consent	Fully informed, Informed consent process compliance^1^	□ Yes □ No □ Can't answer □ Not applicable
Scientificity	Level of evidence	RCT, proper research design, revision of the latest version of the research protocol, transparency of clinical research	□ Yes □ No □ Can't answer □ Not applicable
	Appropriate research method	Proper inclusion/exclusion criteria^1^, study type, definition of primary/secondary endpoints matching research purpose, properly sample size estimation^2^, safety endpoints.	□ Yes □ No □ Can't answer □ Not applicable
	Study protocol dissemination	Study protocol published, study protocol provided through internet.	□ Yes □ No □ Can't answer □ Not applicable

AE, adverse event; SAE, severe adverse event; SOP, standard operation procedure.

1 Are there any inclusion and exclusion criteria in the study protocol. Does the inclusion criteria of prospective study reflect the voluntary participation of subjects in the study. Is the exclusion criteria a simple repetition of the inclusion criteria.

2 Is there any previous research data support for the calculation of sample size in confirmatory research.

### Data source

This study obtained 272 IITs sponsored by Shanghai non-profit organizations in 2015 and 2016, mainly including the standardized application of clinical diagnosis and treatment technology for frequently occurring diseases, chronic diseases, and difficult diseases and the promotion of research achievements. We included IITs that are (1) on human subjects, (2) with protocol attachment or published protocol, and (3) with enrolled patients study. Animal or vitro experiments, projects without data collection, and studies without protocols were excluded. Finally, a total of 257 IITs were included in our research, owing to four projects were excluded due to not human subjects, and another 11 were excluded due to actively apply for project termination.

### Data extraction and quality assessment

Our previous study refers to the risk-based monitoring method advocated by the international community and globally. It explored a set of standard processes in the framework of clinical research and regulation, which mainly included preparation before quality assessment, self-assessment of the project team, centralized inspection, on-site inspection, report writing, and comprehensive assessment. Referring to the clinical research quality assessment process, our team provided mid-term verification and quality control technical support for the quality assessment of the implementation process of IIT projects (21). 257 clinical research projects' quality assessment was also evaluated through the standard processes. Data were collected based on the review of research protocols, ethical approval, case report forms (CRFs), and informed consent submitted by researchers.

A nine-member committee is mainly responsible for data collection and access the indicators of IIT, including PM, DM, CRA, statistician, clinical expert, financial expert, scientific research management expert. PM was responsible for work coordination, DM was responsible for database building and management, two CRA were responsible for the collection of data on quality and ethical regulation, a financial expert was responsible for the collection of data on implementation of the project funds, a senior research manager was responsible for the collection of data on overall progress and enrollment progress of IITs, and the statisticians and clinical experts are responsible for the collection of data on scientific aspects which mainly assess level of evidence and appropriate research method of IITs. All text reviewers were trained in standard procedures to review research files.

For quantitative analysis, the following quality indexes were assessed: overall progress, enrollment progress, budget implementation rate. Each quality index was scored from 0 to 10. Overall progress was divided into not-enrolled, enrolled, treatment completed, follow-up completed, research type including RCT, Prospective cohort, Retrospective study, Real world research, Others. These statuses were given different scores. For quantifying data, each quality index was scored based on the components in research files proportionally. The total score for each index is 10 point. If any “No” and “Can't answer” were answered in each subindex, one point would be deducted. The minimum score of each index is 0.

### Statistical methods

The REDCap [Research electronic data capture (http://projectredcap.org)] was used to input data to control the quality of data. All statistical analyses were performed by R statistical software. Descriptive of basic characteristics of included IITs was presented by mean standard deviation or percentage, as appropriate. The structure validity of the index system of the quality evaluation of clinical research was tested by confirmatory factor analysis. Model fit was evaluated using the Tucker-Lewis Index (TLI), CMIN/DF the ratio chi-square (χ)/degrees of freedom (DF), Goodness-of-Fit Index (GFI), Comparative Fit Index (CFI), Standardized Root Mean Squares Residual (SRMR), and the Root Mean Square Error of Approximation (RMSEA). This index ranged from 0 to 1, the better the fit is, and it is generally believed that CFI and TLI should be >0.9, and GFI is at least >0.80 (22). RMSEA is the index of evaluation model fitting. SRMR index measures the fitting degree of the model by measuring the standardized difference between the observed correlations and the model implied correlations about variables. The closer it is to 0, RMSEA values as high as 0.07 were regarded as acceptable and SRMR should be <0.08 (23). It is considered that the model fits well (22). All statistical analyses were performed by using the R statistical software, “lavaan” package, and “semPlot” package (R version 3.5.3). Two-sided *P* values of < 0.05 were considered to indicate statistical significance. In Excel, the radar plot was generated by using the insert function.

## Results

### The framework of quality indexes for IITs

We conducted two rounds of Delphi panels. The positive coefficient of the two rounds of experts in this study was 100% and the degree of expert authority was 0.932, indicating that the experts participated actively, showing a high degree of authority and a good effect of consultation. We revised some indices by summarizing and analyzing experts' opinions in the first round. For example, study protocol dissemination, one of the scientificity attributes, has been included. Experts believed that all prospective clinical trials should have an appropriate registry before the first participant is enrolled, and study protocol should be made public. At the end of the first round, we deleted two indicators and increased one indicator, and we modified the expression. In the second round of Delphi, we fed back the results of the first round consultation to the experts. Experts reached consensus on the revised indicators during the second round. Furthermore, the CFA method was used to analyze the reliability and validity of the indicators. The research finally identified the conceptual quality indicators for IITs which contain four themes: progress, quality, regulation, and scientificity. As shown in Table 1, 4 quality properties and 13 quality indices were developed through conducting clinical trials.

### Characteristics of included IITs

**Figure 2:**
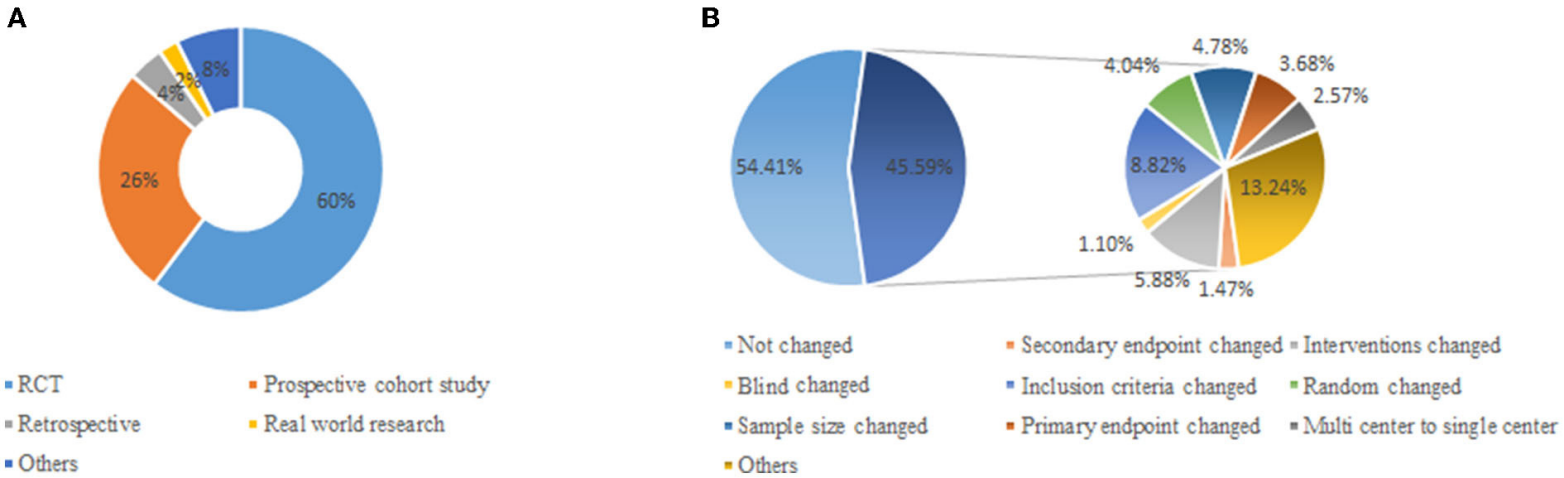
Distributions of research type and protocol change for IITs. **(A)** Distribution of research type for IITs. **(B)** Distribution of protocol change for IITs.

In 2015 and 2016, non-profit government funded 272 IITs, covering 30 tertiary first-class hospitals in Shanghai. It was concluded in 2019, 4 projects were excluded and actively apply for the termination of 11 clinical research projects. Finally, 257 studies were included for validation of quality indicators for IITs: 94.49% (*n* = 272) studies were assessed, 4.04% (*n* = 272) and 1.47% (*n* = 272) studies terminated early (Table 2). 41.54% (*n* = 272) studies were internal medicine, others (18.38%, *n* = 272) mainly included department of facial features, obstetrics and Gynecology, pediatrics. The majority of the studies (60.29%, *n* = 272) were in RCT, others (7.35%, *n* = 272) mainly included diagnostic and cross-sectional study. 58.82% (*n* = 272) of the studies were Single center. 57.35% of the studies had a sample size of 100–500 and only 26.84% (*n* = 272) of the studies had a sample size of <100. In 45.59% of the projects, the research protocol changed during the implementation of the study. As it is shown in Figure 2, 8.82% of trials changed inclusion criteria during the implementation of the study. Interventions and sample size also changed (5.88%, 4.78%).

**Table 2:** Basic characteristics of included for investigator-initiated trials validation of quality assessment tool.

**Items of characteristics**	**Number of projects**	**Proportion (%)**
Total	272	100
**Research status**		
Coincidence study	257	94.49
Terminated	11	4.04
Not applicable	4	1.47
**Project funding amount**		
150–200	38	13.97
80–100	33	12.13
60–80	48	17.65
50–30	119	43.75
15	34	12.5
**Research field**		
Internal medicine	113	41.54
Surgery	109	40.07
Others	50	18.38
**Research type**		
RCT	164	60.29
Prospective cohort	71	26.1
Retrospective study	11	4.04
Real world research	6	2.21
Others	20	7.35
**Number of centers**		
Single center	160	58.82
Multicenter	112	41.18
**Sample size**		
<100	73	26.84
100–500	156	57.35
>500	43	15.81
**Enrollment rate**		
Completed	135	49.63
More than half of the participants	70	25.74
<50% of participants	67	24.63

### Validation of quality assessment tool of IITs

**Figure 3:**
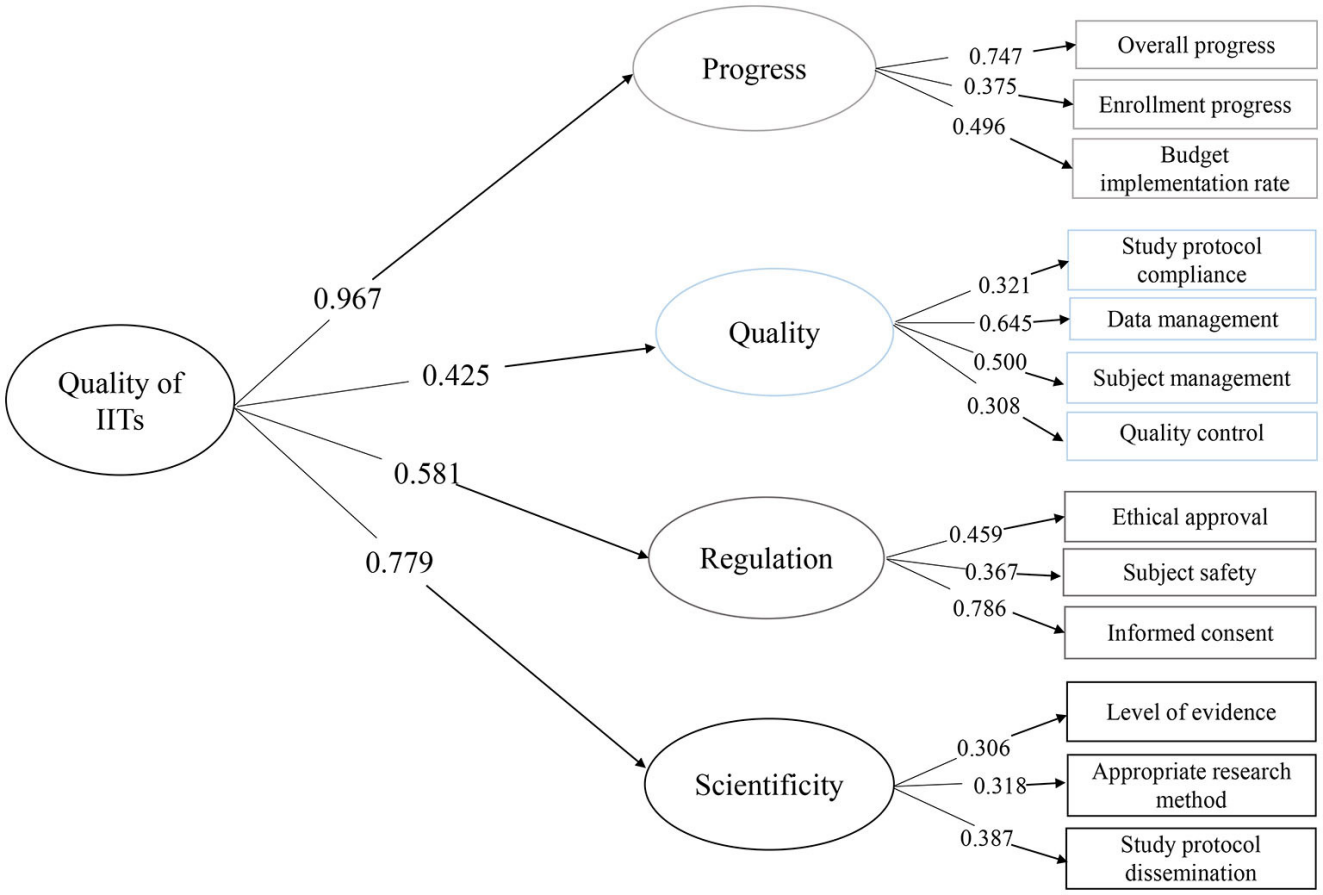
Confirmatory factor analysis (CFA) of key indicators to the quality of IITs.

The preliminary quality indicators of quality assessment tools were further evaluated construct validity by using CFA (Figure 3). The critical quality properties and quality indexes between expertise and CFA are consistent. In this study, CMIN/DF, GFI, CFI, TLI, RMSEA, and SRMR indexes were selected as the indexes of the statistical model. It can be noticed in Table 3 that CMIN/DF was 1.868 <3, GFI, CFI, and TLI were all larger than 0.9, RMSEA was <0.07, and SRMR was <0.08, and it can be considered that the above quality assessment index extraction results were feasible. As is shown in Table 4, factor loadings were most of all *P* < 0.05 and ranged from a minimum of 0.306 to a maximum of 0.786. Meanwhile, the factor loadings between overall IITs quality and one-class index ranged from 0.425 to 0.967 with *P* < 0.05. CFA was used for the index screening of 257 IITs on 17 indices. The results of CFA suggested that the standardized factor loading of 17 indices were all statistically significant, and the factor loading all remained above 0.3 (23), and thus the preliminary entry screening did not delete any index.

**Table 3:** Results of the overall model fitness test for confirmatory factor analysis.

**The dimension**	**CMIN/DF**	**GFI**	**CFI**	**TLI**	**RMSEA**	**SRMR**
Cognitive ability	1.868	0.916	0.936	0.919	0.063	0.076
Demonstrating compliance	Up to standard	Up to standard	Up to standard	Up to standard	Up to standard	Up to standard

**Table 4:** Validation of quality assessment tool of investigator-initiated trials by CFA.

**Quality properties**	**Quality indexes**	**Estimate**	**Factor load**	**SE**	***z*** **-value**	***P*** **-value**
Progress	Overall progress	1.000	0.747			
	Enrollment progress	0.452	0.375	0.209	2.159	0.031
	Budget implementation rate	0.583	0.496	0.254	2.266	0.023
Quality	Study protocol compliance	1.000	0.321			
	Data management	0.633	0.645	0.209	3.030	0.002
	Subject management	0.590	0.500	0.192	3.077	0.002
	Quality control	0.350	0.308	0.129	1.160	0.046
Regulation	Ethical approval	1.000	0.459			
	Subject safety	0.342	0.367	0.073	1.936	0.043
	Informed consent	2.619	0.786	0.886	2.995	0.003
Scientificity	Level of evidence	1.000	0.306			
	Appropriate research method	0.352	0.318	0.216	1.629	0.047
	Study protocol dissemination	1.365	0.387	0.843	1.638	0.038
Overall quality						
	Progress	4.149	0.967			
	Quality	0.133	0.425	0.124	1.576	0.033
	Regulation	0.213	0.581	0.114	1.876	0.023
	Scientificity	0.131	0.779	0.118	1.917	0.013

### Results of quality assessment of IITs

**Figure 4:**
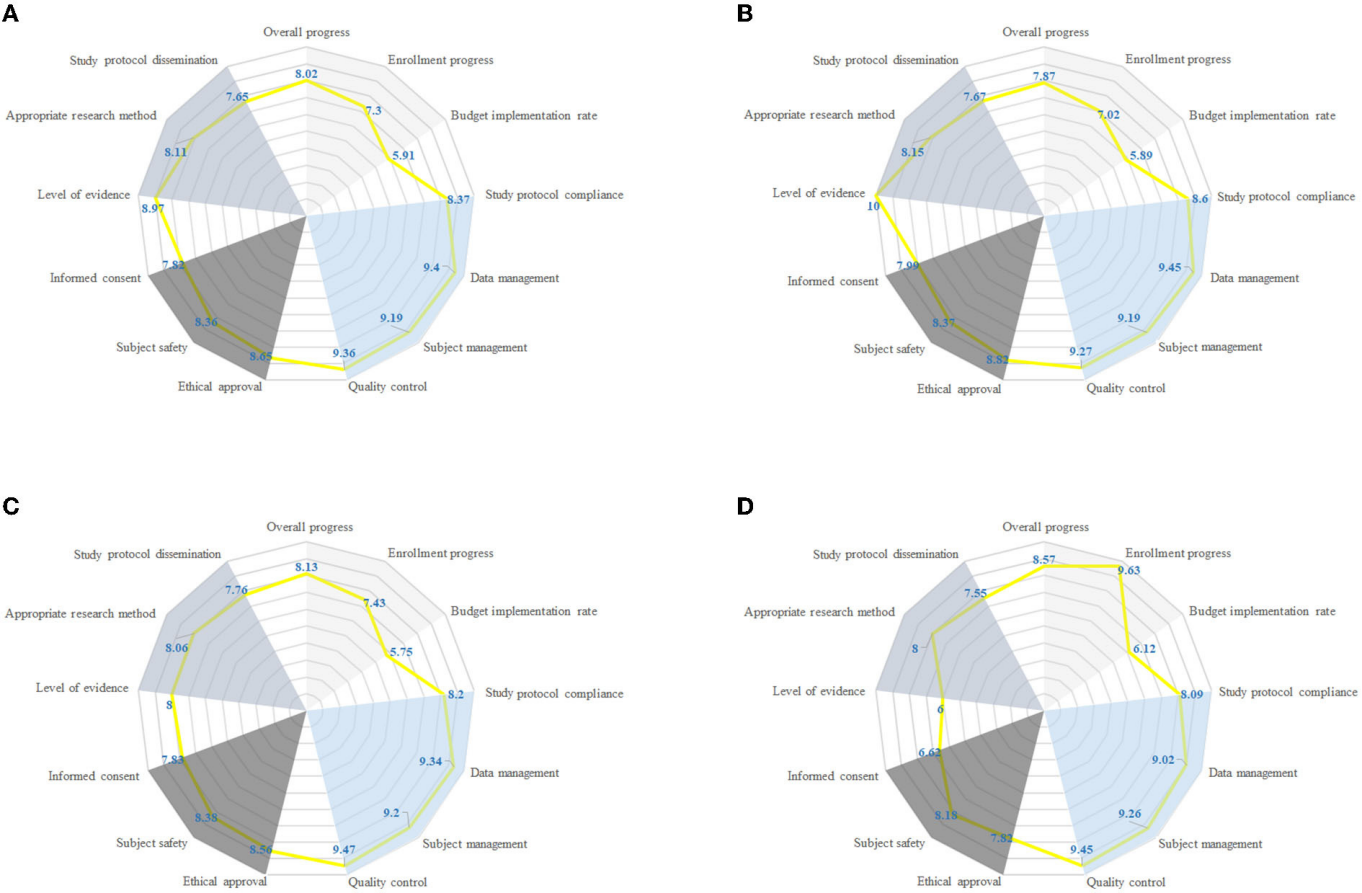
Radar chart comparing different research type with respect to 13 quality characteristics (individual radial axes). Each axis shows fraction of IITs with given property, such as overall progress, enrollment progress, budget implementation rate. **(A)** Quality characteristics by all IITs. **(B)** Quality characteristics by RCT. **(C)** Quality characteristics by prospective cohort. **(D)** Quality characteristics by retrospective study.

Radar graphing was used to display data. Radar plots had a series of spokes or rays arising from a central point, with each ray showing a different index, such as data management. Figure 4 illustrated that polygons were created with each spoke showing one of the secondary indexes and each point on the spoke reflecting the magnitude of the mean results, and different colors region represent the different one-class index. Among 257 IITs, data management was well-presented with an average score of 9.40, followed by quality control of 9.36. Enrollment progress and budget implementation rate scored low, which were 7.30 and 5.91, respectively. RCT, prospective cohort and retrospective performed similarly in subject management, appropriate research method, and quality control. However, RCT had lower scores than prospective cohort and retrospective study in enrollment progress (7.02 vs. 7.43, 9.63, respectively).

## Discussion

Our study proposed a proactive quality assessment consideration for IITs which consisted of four aspects: progress, quality, regulation, and scientificity. A total of 257 IITs were included for the validation of quality indicators. We further confirmed the structural validity of the critical quality properties and quality indexes as latent variables. According to the results of CFA, CMIN/DF, GFI, CFI, TLI, RMSEA, SRMR, and other indicators used in the model test all met the requirements. The CFA model revealed strong positive links from four quality indexes to the overall quality of ITTs.

The panorama quality assessment tool can effectively evaluate the quality of research, and help find major bias. For the management side like National Institutes of Health, the assessment tool can effectively improve the efficiency and effectiveness of funding. With the help of this tool, funding agencies can better decide which project should be supported or terminated. Assessment tool is very important to manage and improve the quality of clinical trials. Quality assessment standard processes in the framework of clinical research and regulation follow international guidelines. Traditionally, the International Conference on Harmonization–Good Clinical Practices (ICH-GCP) described two verification activities: quality control and quality assurance, with the aims to protect the rights and wellbeing of subjects and ensure protocol compliance and data integrity. FDA (17) and EMA (18) guidelines both issued in 2013, ICH GCP guidelines (16) issued in 2016 and NMPA GCP (24) issued in 2020 suggested focusing on critical data and critical processes, and encouraged to adopt risk-based approaches to monitor clinical trials. A series of research studies reported that risk-based monitoring had the potential to make trials more efficient and reduced costs (25, 26). In this study, risk-based approach was adopted to identify critical data of IITs and improve the capacity of self-regulate overall quality. Various research types of IIT projects will increase the difficulty of quality evaluation. Standardization of practices in monitoring activities will be a suitable method for the management of IITs.

We further confirmed the structural validity of the IITs quality assessment tools by CFA. The model also presented the importance of progress and scientificity. Progress, measured jointly by overall progress, enrollment progress, budget implementation rate, exerted direct and indirect effects on the overall quality of IITs in our theory which was confirmed by the model. Poor recruitment of participants is the most common reason for the RCT discontinuation, which reflects a large waste of scarce research resources (27). In this study, enrollment progress and budget implementation rate scored 7.30 and 5.91 respectively, which were rather low. The reason for the low recruitment progress of subjects may be linked with funding, design, recruiter, or participant (28). In addition, there were differences in the progress of subject recruitment among different research types, and the lowest recruitment progress is RCT. Scientificity, measured jointly by level of evidence, appropriate research method, and study protocol dissemination had direct and indirect impacts on the overall quality of IITs in our theory which was confirmed by the model. Our study found that almost half (117/257) of the research protocol adjusted during the implementation process may be associated with poor design. It is important that all research findings, including negative and inconclusive results are reported transparently and made publicly available in order to avoid unnecessary duplication of research or biases in the clinical knowledge base (8). While our study found that 80% of the project research protocols have not been published in public journals or websites, which was consistent with the literature (29). Also, poorly conducted research may result in slow dissemination of research results which has been reported among registered clinical studies (29), with almost half of the studies remaining unpublished years after completion may be aroused by “a lack-of-time or low priority,” followed by “results not important enough” and “journal rejection” (30).

Different from previous assessment tools like RACT, which is more suitable for ISTs. The panorama tool developed in this study was optimized for funding agencies to assess the quality of ongoing IITs. For ISTs, the responsibility to avoid failure due to unsatisfactory progress or scientificity is mostly up to the industrial sponsor. Therefore, the regulatory department can focus on the ethics and quality aspects of the studies. However, for IITs, funding agencies should take progress and scientificity into consideration that the resources will be used more efficiently. Therefore, unlike the traditional point that the most important aspects of clinical trials are subject safety and rights plus data quality, this tool also emphasized the importance of progress and scientificity. Also, the tool can be used to assess the quality of both study design and implementation, and can be used throughout the whole clinical research, regardless of the research types. What's more, a little different from other risk based monitoring tools, this tool not only can find risks and determine the monitoring methods, also can be used to compare the quality of several clinical studies.

Our study had several advantages and limitations as well. First, the samples used to confirm the CFA were collected from ongoing studies, and no previous research was identified to discuss the ongoing studies. Our study developed the tool can objectively reflect the current research status, regardless of the research types. Second, the sample size was up to standard. We collected more than 200 samples for CFA and while CFI is a non-centrality parameter-based index designed to overcome the limitation of sample size effects (31). Third, our study adopted a risk-based monitoring method to identify critical data and processes, which was in line with international trends and saved resources. The quality assessment tool for IITs enabled us to evaluate the overall quality of IITs and helped refine quality practices in IITs. However, we only included the clinical research projects in Shanghai hospitals. Further research is needed to confirm this tool in more general scenarios.

## Conclusion

The results of critical quality properties and quality indexes between expertise and confirmatory factor analysis were basically consistent, indicating applying this panoramic quality assessment tool for overall quality evaluation of IITs is feasible and validated. This panorama tool can enable project management departments to effectively and dynamically manage the quality of their studies, and can timely and dynamically find errors, take actions to prevent major bias. Furthermore, the project management departments will be able to terminate the “low-quality” project in advance, and provide rolling support for the “high quality” project based on the situation of the projects. It is hoped that this tool can provide project management departments with resources for effective and dynamic management of researches and avoid waste of resources, as well as a manner to improve the quality of IITs in the future.

## Data availability statement

The original contributions presented in the study are included in the article/supplementary material, further inquiries can be directed to the corresponding author.

## Author contributions

WL, TH, WZ, and BQ designed the study. WZ and BQ co-ordinated the study. WL, TH, JJ, TQ, ES, JD, and XM performed the acquisition of data and the statistical analysis. WL and TH drafted the manuscript. All authors revised the final manuscript and approved this version to be published.

## Funding

This work was supported by the Project of Shanghai Jiao Tong University School of Medicine (Grant No. WK2003), Shanghai Jiao Tong University Medical and Industrial Cross Project (YG2022QN004), and Program of Shanghai Academic/Technology Research Leader (Grant No. 21XD1402600).

## Conflict of interest

The authors declare that the research was conducted in the absence of any commercial or financial relationships that could be construed as a potential conflict of interest.

## Publisher's note

All claims expressed in this article are solely those of the authors and do not necessarily represent those of their affiliated organizations, or those of the publisher, the editors and the reviewers. Any product that may be evaluated in this article, or claim that may be made by its manufacturer, is not guaranteed or endorsed by the publisher.

## Footnotes

^1^ https://www.tmf-ev.de/ADAMON_EN/Projectdescription.aspx

## Abbreviations

AE: adverse event
CFI: comparative fit index
GCP: good clinical practice
ICH: International Conference on Harmonization
PQRS: progress-quality-regulation- scientificity
RMSEA: root mean square error of approximation
NMPA: National Medical Products Administration
SAE: severe adverse event
SEM: structural equation modeling
SOP: standard operation procedure
TLI: Tucker-Lewis index.
